# A repetitive elements perspective in Polycomb epigenetics

**DOI:** 10.3389/fgene.2012.00199

**Published:** 2012-10-08

**Authors:** Valentina Casa, Davide Gabellini

**Affiliations:** ^1^Division of Regenerative Medicine, Stem Cells, and Gene Therapy, Dulbecco Telethon Institute and San Raffaele Scientific InstituteMilano, Italy; ^2^Università Vita-Salute San RaffaeleMilano, Italy

**Keywords:** repeats, Polycomb, non-protein-coding RNA, nuclear structure, FSHD muscular dystrophy

## Abstract

Repetitive elements comprise over two-thirds of the human genome. For a long time, these elements have received little attention since they were considered non-functional. On the contrary, recent evidence indicates that they play central roles in genome integrity, gene expression, and disease. Indeed, repeats display meiotic instability associated with disease and are located within common fragile sites, which are hotspots of chromosome re-arrangements in tumors. Moreover, a variety of diseases have been associated with aberrant transcription of repetitive elements. Overall this indicates that appropriate regulation of repetitive elements’ activity is fundamental. Polycomb group (PcG) proteins are epigenetic regulators that are essential for the normal development of multicellular organisms. Mammalian PcG proteins are involved in fundamental processes, such as cellular memory, cell proliferation, genomic imprinting, X-inactivation, and cancer development. PcG proteins can convey their activity through long-distance interactions also on different chromosomes. This indicates that the 3D organization of PcG proteins contributes significantly to their function. However, it is still unclear how these complex mechanisms are orchestrated and which role PcG proteins play in the multi-level organization of gene regulation. Intriguingly, the greatest proportion of Polycomb-mediated chromatin modifications is located in genomic repeats and it has been suggested that they could provide a binding platform for Polycomb proteins. Here, these lines of evidence are woven together to discuss how repetitive elements could contribute to chromatin organization in the 3D nuclear space.

## Introduction

In the last decade, when the genomic sequences of *Homo sapiens* and several model organisms became available, there was the realization that the number of protein-coding genes does not correlate with organism complexity. In fact, worms or flies have approximately the same number of protein-coding genes as mice or humans (Taft et al., 2007). On the other hand, the non-protein coding component of the genomic DNA, and in particular repetitive elements, represent a progressively larger proportion of the genome in organisms with increasing complexity (Neguembor and Gabellini, 2010). Recent estimations indicate that repetitive sequences could account for up to 66–69% of the human genome (De Koning et al., 2011). While this strongly suggests that it might significantly contribute to higher eukaryotes sophistication, the repetitive fraction of the genome is largely ignored.

The advent of next generation sequencing (NGS) has permitted a genome-wide view to gene expression and chromatin structure. However, NGS-based studies often take into account only reads for which a unique genomic alignment can be obtained, thus discarding data deriving from repetitive DNA (Myers et al., 2011). Despite this, there is increasing evidence of the peculiar functions of the repeated (epi)genome. For example, the role of DNA repeats in chromosome structural organization, gene regulation, genome integrity, and evolution has been described (Kidwell and Lisch, 2000; Lander et al., 2001; Waterston et al., 2002; Feschotte, 2008; Ting et al., 2011; Zhu et al., 2011).

DNA repeats can be also transcribed, frequently in a cell and tissue-specific fashion. Analyses based on Cap Analysis of Gene Expression (CAGE) technology from the Functional Annotation of Mouse (FANTOM) project, revealed an unexpectedly large proportion of capped-transcripts initiating from repetitive units. It has been suggested that these can provide regulatory elements to protein-coding genes, such as alternative promoters, exons, or polyadenylation sites, and ncRNAs, thus significantly expanding the regulatory capability of higher eukaryote genomes (Wang et al., 2007; Bourque et al., 2008; Faulkner et al., 2009; Tyekucheva et al., 2011). Moreover, binding sites for important regulatory factors such as CTCF or TP53 are often associated with genomic repeats (Wang et al., 2007; Bourque et al., 2008; Chadwick, 2008; Simeonova et al., 2012).

Repetitive elements can either mobilize or rearrange in somatic tissues, thus providing an unexpected dynamic dimension to the normal physiology of the soma, but also contributing to the etiopathogenesis of diseases (Kazazian et al., 1988; Ting et al., 2011; Zhu et al., 2011). For the role they can play in genome plasticity, repeats need to be finely tuned. To accomplish this, epigenetic mechanisms including RNA interference (RNAi), DNA methylation, and histone modifications are used to deal with the potentially dangerous effects of repeat transpositions and rearrangements (Slotkin and Martienssen, 2007; Maksakova et al., 2008).

Polycomb group proteins (PcG) are epigenetic repressors with the important function of maintaining the memory of transcriptional programs during development and differentiation (Morey and Helin, 2010; Schuettengruber et al., 2011). However, PcG role appears to go far beyond gene regulation, as they have been associated with many other important nuclear processes, including the regulation of higher order genome architecture and structure (Bantignies and Cavalli, 2011). Importantly, the vast majority of mammalian PcG proteins bind to non-coding DNA, and in particular repetitive elements, which for their intrinsic feature of being present in several copy number, may constitute binding platforms for Polycomb binding in mammals (Cabianca et al., 2012).

In this review, the biological role of DNA repeats and their epigenetic regulation is summarized with the hope of fostering new investigations of this largely unexplored region of the human genome.

## Genetics and epigenetics of repetitive elements

Using classical annotation processes, about 50% of a typical mammalian genome is annotated as DNA repeats, 5–10% as genes and functional elements and the remaining 40–45% as DNA of unknown function. One caveat with traditional repeat annotation is that DNA repeat identification approaches, e.g., the RepeatMasker program (Smit, 1996–2004), use well-curated libraries of known repeat family consensus sequences. By doing so, ancient or divergent DNA repeat classes fail to be identified as repeats. Recently, using a highly sensitive alternative strategy, it was predicted that there may be more than 840 Mbp of additional repetitive sequences in the human genome, thus suggesting that up to 70% of the total genome is composed of repeats (De Koning et al., 2011).

DNA repeats can be present in different arrangements and sizes: they can be widely interspersed repeats (Table 1) or they can be located one next to another to form tandem repeats (Table 2). Repeats can range in size from 1 to 2 bases to millions of bases and might comprise just two copies or millions of copies (Batzer and Deininger, 2002; Jurka et al., 2007; Kim et al., 2008; Britten, 2010; Hua-Van et al., 2011).

**Table 1:** **Major features of the most represented interspersed repetitive elements in the human genome**.

**Repeat type**				**Estimated number of copies**	**Average length**	**Mobility**	**Estimated % genome coverage**	
Interspersed	Retrotransposons	LTR	LTR (Long terminal repeat) or ERV (Endogenous retroviruses) (MaLR, ERV, ERV1, ERV-K, ERV-L, etc.)	200,000	6–11 kb	Autonomous retrotransposition (retroviral-like)	8%	42%
		Non-LTR	LINE (Long interspersed element) (L1, L2, CR1, etc.)	500,000	6 kb	Autonomous retrotransposition	20%	
			SINE (Short interspersed element) (Alu, MIR, etc.)	1,000,000	0.3 kb	L-1 dependent Retrotransposition	13%	
			SVA SINE-R/VNTR/Alu	2700	2–5 kb	L-1 dependent Retrotransposition		
	DNA transposons	DNA transposons (MER1, MER2, Mariner, Merlin, etc.)		300,000	1–3 kb	inert	2–3%	

Number of copies and genome coverage are estimated values based on current genome coverage.

**Table 2:** **Major features of the most represented tandem repeats in the human genome**.

**Repeat type**			**Unit length**	**Array length**	**Estimated % genome coverage**
Tandem	Satellite	Alpha-Satellite	171 bp	3–5 Mb	22–25%
		Satellite II (HsatII)	23–26 bp or multiple	10–70 kb	
		Satellite III (GAATGn- simple sequence)	5 bp or multiples up to 70 bp	7.5–100 kb		
		Beta-Satellite	68 bp	2–14.5 kb		
		Gamma-Satellite	220 bp	10–200 kb	
	VNTR (Variable number of tandem repeats)	Microsatellite (Short tandem repeat)	1–13 bp	Hundreds bp	
		Minisatellite (including telomeric repeats)	6–100 bp	1–15 kb or more	
		Macrosatellite	2–12 kb or more	Tens up to hundreds bp	

Genome coverage is estimated on the basis of current genome coverage.

### Interspersed repeats

Interspersed repeats are the results of ancient or present activity of mobile genetic elements. These elements can mediate their own mobilization either by a cut-and-paste mechanism, as DNA transposons, or by a copy-and-paste process, like retro-transposons (Solyom and Kazazian, 2012). While DNA transposons are now considered immobile, some retro-transposon elements are able to mobilize themselves and other elements. Retrotransposons are composed of long terminal repeat (LTR) and non-LTR containing elements. The LTR retrotransposons are endogenous retroviruses (ERVs) that have lost the ability to go outside the host cell due to a non-functional envelope gene. Non-LTR retrotransposons can be subdivided into long interspersed elements (LINEs), short interspersed elements (SINEs) and, in hominid genomes, medium sized SVAs (SINE-R/VNTR (variable number of tandem repeat)/Alu). In humans, the most important LINE is the RNA polymerase II transcribed LINE-1 (L1), while SINEs are essentially represented by the RNA polymerase III transcribed Alus. L1 is the only element able to encode the proteins required for mobilization. Hence, these are the only known autonomously active human retrotransposons. L1s are also responsible for the mobilization of the non-autonomous Alus, SVAs and processed pseudogenes (cellular mRNAs that become substrates of the reverse transcriptases and are inserted into the genome).

Mobile elements have a significant role in evolution and in generating genetic diversity. For example, the genome fraction occupied by mobile elements varies in different species and each eukaryote displays a specific mobile element complement, suggesting that mobile elements are important players during speciation and evolution (Faulkner, 2011). Being significant contributors to the copy number variation present in humans, mobile elements are also an important source of genetic variation (Brouha et al., 2003; Bennett et al., 2004; Mills et al., 2007; Iskow et al., 2010; Ekram et al., 2012). Moreover, mobile elements can display differential activity in different tissues of the soma, suggesting that every individual is a genetic mosaic variegated by the differential insertion of mobile elements (Muotri et al., 2005, 2010). Finally, retrotransposons have recently been identified as a major source of epigenetic variations in the mammalian genome (Ekram et al., 2012). Retrotransposition, with only few exceptions such as V(D)J recombination (Brack et al., 1978), is an almost unique source of somatic genetic mosaicism, leading not only to heritable genetic variation but also to intra-individual variability. This represents a revolutionary concept that is changing the view of this class of repetitive elements (Faulkner, 2011).

Due to their nature, mobile elements have the potential to affect common diseases, through structural variation, deregulated transcriptional activity or epigenetic effects. Moreover, their transposition can directly cause insertional mutagenesis, as proved by the existence of nearly 100 examples of mobile element insertions causing disease (Lee et al., 2012; Solyom and Kazazian, 2012).

For their genotoxic potential, mobile elements are usually kept repressed by epigenetic mechanisms. DNA methylation represents one of the major players in the repression of repetitive elements (Liang et al., 2002; Kato et al., 2007). A recent study performed a comprehensive genome-wide methylation analysis on all repetitive elements in human embryonic stem cells and fetal fibroblasts (Su et al., 2012). Among all classes of repetitive elements, LINE, LTR, DNA transposon, and also satellite tandem DNA repeats appear more susceptible to changes in DNA methylation, thus suggesting that they are specifically regulated and silenced during cellular differentiation. Importantly, transposon-free regions (TFRs) in the genome have been selectively conserved and are associated with regions including CpG islands, suggesting that in mammalian genomes there are fragments of DNA that are largely unable to tolerate transposon insertion (Simons et al., 2006).

Aberrant repetitive DNA methylation can be associated with diseases. For example, hypo-methylation of L1, Alu, LTR, but also of satellite repeats, is significantly associated with tumor progression in multiple cancers such as gastrointestinal stromal tumors, myeloma, and lung cancer (Rauch et al., 2008; Bollati et al., 2009; Igarashi et al., 2010).

Additionally, mobilization of L1 repeats has been associated with both physiological and pathological processes and is regulated by DNA methylation (Muotri et al., 2010). L1 mobilization has been associated with brain cell development, where the occurrence of L1 retrotransposition in adult cells has been suggested to contribute to neuronal somatic diversification (Muotri et al., 2005). This mechanism, so far assigned specifically to human neural progenitors and adult hippocampus, is modulated by the methyl-CpG-binding protein 2 (MeCP2) (Muotri et al., 2005, 2010; Coufal et al., 2009). Importantly, in RETT syndrome, a mental retardation disorder caused by mutation in the *MECP2* gene, an extensive de-regulation of L1 retrotransposition in neurons has been reported (Muotri et al., 2010; Solyom and Kazazian, 2012).

Besides DNA methylation, several repressive histone modifications, including H3K9me3, H3K27me3, and H4K20me3, are also enriched on interspersed repeats (Martens et al., 2005; Mikkelsen et al., 2007; Leeb et al., 2010). Importantly, a re-estimation of chromatin immunoprecipitation results on repetitive elements from high-throughput sequence data of human and mouse cells has been recently conducted (Day et al., 2010). According to this analysis, different members of the murine ERV family of repeats appear to assume distinct patterns of histone modifications, which are representative of a specific pattern of heterochromatin formation. While transposable elements belonging to ERV-K and ERV1 subfamilies are enriched for histone marks typical of constitutive heterochromatin such as H3K9me3 and H4K20me3 in mouse ES cells, ERV-L and MaLR families are characterized by the hallmark of Polycomb-mediated silencing H3K27me3 (Mikkelsen et al., 2007; Dong et al., 2008; Day et al., 2010).

A remarkable finding from these studies is that silencing of repetitive elements can be redundant and flexible. This has been shown by independent groups and within independent silencing pathways. For example, during the stages of global DNA de-methylation in early embryonic mouse development, the RNA-interference guardian machinery become responsible for controlling the expression of intracisternal A particle (IAP), ERV-K, and ERV-L retrotransposons, thus preserving genome integrity (Svoboda et al., 2004). Additionally, studies of mouse ES cells deficient for the H3K9 histone methyltransferases Suv39h showed that decreased H3K9me3 levels in the repetitive elements were compensated by increases in H3K27me3 enrichment (Peters et al., 2003). Thus, different and largely independent repression pathways can converge and compensate each other’s function. Most likely, this has to do with the necessity of the cells to guarantee multiple levels of protection from aberrant activation of mobile elements.

Overall, the epigenetic repression of repetitive elements on one hand prevents dramatic nuclear effects such as genotoxicity, but on the other hand allows the specific regulation of such elements occurring in the germ line (Peaston et al., 2004), embryonic cells (Kano et al., 2009) and, perhaps to a lesser extent, during later developmental phases (Muotri et al., 2005, 2010).

### Tandem repeats

Tandem repeats constitute a large portion of the human genome, and account for a significant amount of its copy number variation (Warburton et al., 2008). Besides their role in evolution (Warburton et al., 1996; Rudd et al., 2006; McLaughlin and Chadwick, 2011), they have been found to be critical in several other processes, including heterochromatin formation, chromosome segregation, (Morris and Moazed, 2007) and X-chromosome inactivation (XCI) (Chadwick, 2008). Moreover, repeat instability is at the basis of a number of diseases (Lopez Castel et al., 2010).

Tandem DNA repeats in the human genome show a wide range of unit sizes, spanning from a few base pairs in microsatellites, to several kilobases in megasatellites (Gelfand et al., 2007; Ames et al., 2008; Warburton et al., 2008). At a given locus, the tandem repeat copy number is usually polymorphic among individuals, and for this reason they are more commonly known as variable number tandem repeats (VNTRs).

One of the principal families of DNA tandem repeats in the genome is represented by the satellite DNA of chromosome centromeres. Indeed, maintenance of the structural integrity of centromeres and telomeres is one of the most important functions of tandem repeats (Blackburn, 1984). Centromeres have the fundamental role to ensure proper chromosome segregation during cell division. In the human genome, they consist of several Mb of alpha-satellite DNA, which is composed of a 171 bp repeat unit. Chromosome-specific higher-order repeat structures are typical of this type of repeat, as they are important for centromere function (Schueler et al., 2001). Forms of higher-order organization have also unexpectedly been characterized in “simple satellite” sequences such as GAATGn and VNTRs (Warburton et al., 2008), but whether this bears functional relevance has yet to be determined.

For their function, centromeres of higher eukaryotes require an epigenetic specification, rather than a defined DNA sequence. Indeed, centromeric regions localize in the pericentric heterochromatic domain of the interphase nucleus, and they are enriched in H3K9me3, H4K20me3, H3K27me1 histone marks (Peters et al., 2001, 2003; Guenatri et al., 2004; Martens et al., 2005; Mikkelsen et al., 2007; Dong et al., 2008) and in proteins like the centromere-specific H3 variant Centromere protein A (CENP-A) (Yoda et al., 2000; Lo et al., 2001; Blower et al., 2002). As already described for the epigenetic regulation of interspersed repeats, loss of the H3K9 histone methyltransferases (HMTases) Suv39h, which are responsible for the tri-methylation of H3K9 (Peters et al., 2003), activates a compensatory mechanism leading to increase in H3K27me3 (a hallmark of Polycomb-mediated silencing). This underscores an unexpected plasticity between the H3K9 and H3K27 methylation systems (Peters et al., 2003).

In mice, where two different types of repetitive DNA sequences are associated with centromeres, major satellite repeats (6 megabases of 234 bp units) in the pericentromeric region, and minor satellite repeats (600 kb of 120 bp units) in the centromeric region (Choo, 1997), two distinct heterochromatic domains are distinguishable, which became important signatures of mouse interphase nuclei (Guenatri et al., 2004). Pericentromeric satellite DNA of different chromosomes forms large heterochromatic clusters, which upon DAPI staining result in DAPI-dense structures called chromocenters. These formations are typically enriched for the heterochromatin protein 1 alpha (HP1α). The minor satellite DNA, instead, forms individual heterochromatin structures containing the CENP proteins (Guenatri et al., 2004).

In the human genome, the main groups of tandem repeats are the micro-, mini- or macro-satellites (Warburton et al., 2008). They are highly polymorphic in the general population and for this reason they are widely used as genetic markers. Macrosatellites consist of arrays of 1–12 kb repeat units, with a number of repeats ranging from a few to over one hundred (Warburton et al., 2008; Moseley et al., 2012). They can be either chromosome specific, as DXZ4 at chromosome Xq23 (Giacalone et al., 1992) and ZAV at chromosome 9q32 (Tremblay et al., 2010) or they can be associated with two or more chromosomal locations, such as D4Z4, on chromosomes 4q35 and 10q26; (Deidda et al., 1995; Winokur et al., 1996) and RS447, on 4p15 and 18p23; (Gondo et al., 1998).

DXZ4 and D4Z4 macrosatellites are both extensively regulated at the epigenetic level, and they have been described as being associated with either euchromatic or heterochromatic states. Contraction of the 3.3 kb polymorphic D4Z4 tandem repeat array on chromosome 4q35 is associated with facioscapulohumeral muscular dystrophy (FSHD) where a shortening below the threshold of 11 repeat units generates an epigenetic and topologic remodeling of the locus, thus leading to the pathology (Cabianca and Gabellini, 2010). The X-linked DXZ4 macrosatellite locus, instead, has an opposing conformation to that of the surrounding chromosome, constituting a euchromatic dot in the inactive X chromosome, and vice versa (Chadwick, 2008). For their very peculiar epigenetic features and for their involvement in fundamental biological and pathological processes, D4Z4 and DXZ4 could emerge as paradigms for understanding the epigenetic regulation of tandem DNA.

### D4Z4 and DXZ4

Two of the most extensively investigated macrosatellites are the X-linked DXZ4 and the chromosome 4-linked D4Z4. Despite lacking sequence similarity, D4Z4 and DXZ4 macrosatellites share several common aspects (Chadwick, 2009). DXZ4 and D4Z4 are extremely GC rich and belong to a family of human macrosatellites that are noncentromerically located (Giacalone et al., 1992; Kogi et al., 1997; Chadwick, 2009; Tremblay et al., 2010).

Each DXZ4 unit is 3.0 kb long and organized in a tandem array containing 12 to more than 100 copies, localized at Xq23 (Giacalone et al., 1992). As typical for an X-linked locus, DXZ4 is hemizygous in males and subject to XCI in females. However, DXZ4 adopts an opposite chromatin conformation compared to that of the surrounding X chromosome. In males and on the active X-chromosome (Xa), DXZ4 displays features of constitutive heterochromatin, like enrichments in the repressive histone mark H3K9me3, high levels of DNA methylation and association with heterochromatin protein 1 gamma (HP1γ). On the contrary, in the inactive X (Xi), DXZ4 is characterized by euchromatic histone marks such as H3K4me2 and H3K9Ac, a low level of DNA methylation, and is bound by the chromatin regulators CTCF and YY1 (Chadwick, 2008; Filippova, 2008; Moseley et al., 2012). Notably, these features of DXZ4 are remarkably similar to those of the mouse X-inactivation center (Xic), a region of the X chromosome required for XCI (Courtier et al., 1995; Chao et al., 2002; Boumil et al., 2006; Donohoe et al., 2007). Finally, DXZ4 resides at the distal edge of a heterochromatic region targeted by PcG epigenetic repressors (Chadwick and Willard, 2004; McLaughlin and Chadwick, 2011).

The D4Z4 macrosatellite maps to the subtelomeric region of the chromosome 4 long arm, in 4q35. Each unit is 3.3 kb and is present in 11 to 100–150 copies in the general population. Interestingly, reduction of D4Z4 copy number below 11 units is associated with FSHD, one of the most important forms of muscular dystrophy (Wijmenga et al., 1992; Van Deutekom et al., 1993). D4Z4 belongs to a family of repeats with high sequence identity present also in human chromosomes 10q26, 1p12, and the p-arm of acrocentric chromosomes (Lyle et al., 1995; Winokur et al., 1996). This results in frequent exchanges between the 4q35 and 10q26 arrays, which share the highest identity (Van Deutekom et al., 1993). Like DXZ4, D4Z4 is bound by the epigenetic factor YY1 (Gabellini et al., 2002) and displays alternative epigenetic states that parallel the ones of DXZ4 in Xa versus Xi. For D4Z4, the epigenetic make-up is copy number-dependent. The non-contracted array, which retains more than 11 D4Z4 units, displays heterochromatic features like the repressive histone marks H3K9me3 (Zeng et al., 2009) and H3K27me3 (Bodega et al., 2009; Cabianca et al., 2012), histone hypoacetylation (Jiang et al., 2003), as well as a high level of DNA methylation (Van Overveld et al., 2003). Reduction of D4Z4 copy number below 11 units is associated with reduced levels of repressive histone marks (Bodega et al., 2009; Zeng et al., 2009; Cabianca et al., 2012), acquisition of the activating histone marks H3K4me3 and H3K36me2 (Cabianca et al., 2012), DNA hypomethylation (Van Overveld et al., 2003), binding of CTCF (Ottaviani et al., 2009) and loss of Polycomb silencing (Cabianca et al., 2012).

Like DXZ4, D4Z4 is bi-directionally transcribed to generate non-protein-coding RNAs (ncRNAs) (Chadwick, 2008; Snider et al., 2009; Tremblay et al., 2011; Block et al., 2012; Cabianca et al., 2012). In particular, D4Z4 generates a long, chromatin-associated ncRNA (*DBE*-T) selectively in FSHD patients. *DBE-T* functions *in cis* by recruiting the Trithorax protein ASH1L to the FSHD locus leading to chromatin remodeling and de-repression of 4q35 genes (Cabianca et al., 2012). Hence, similarly to the dichotomous behavior observed for DXZ4 on Xi and Xa chromosomes, for D4Z4 the FSHD pathogenesis underlies a major epigenetic switch from a Polycomb repressed state to a Trithorax de-repressed state.

The last, most telomeric D4Z4 unit at 4q35 encodes for a protein called DUX4 (double homeobox 4), which represents one of the major candidates for FSHD (Lemmers et al., 2010). The *DUX4* gene itself originates from a repetitive element, as it is a processed pseudogene of the ancestral *DUXC* gene. Interestingly, *DUX4* and not *DUXC* has been selectively retained in the primate lineage (Clapp et al., 2007; Leidenroth and Hewitt, 2010). In healthy subjects *DUX4* is expressed only in the germ line, while it is epigenetically silenced in somatic tissues (Snider et al., 2010). In FSHD, *DUX4* is aberrantly expressed in skeletal muscle (Dixit et al., 2007; Snider et al., 2010).

DUX4 protein is a transcriptional activator able to bind and activate transcription of MaLR repetitive elements (Geng et al., 2012). Interestingly, MaLR retrotransposons are known Polycomb targets (Day et al., 2010). Hence, DUX4 could have the physiological role of collaborating with Polycomb for the regulation of repetitive elements during early developmental stages and in the germ line.

### Polycomb

PcG proteins and their functional counterpart, the Trithorax Group (TrxG) proteins, are evolutionary-conserved chromatin regulatory factors that were originally identified in *Drosophila* (Schuettengruber et al., 2007, 2011; Morey and Helin, 2010). PcG and TrxG are essential for cellular identity and differentiation in multicellular organisms. Their activity is required to maintain an “epigenetic memory” of specific gene expression patterns. This is at the basis of the establishment of the correct spatio-temporal regulation of gene expression and, more importantly, of its transmission throughout cell division and cell fate choices. In general, PcG collaborates with transcriptional repressors to maintain gene silencing while TrxG works by counteracting PcG activity allowing, if the appropriate transcriptional activators are available, for gene activation (Schuettengruber et al., 2007). In vertebrates, PcG and TrxG play a central role in stem-cell plasticity and renewal, proliferation, genomic imprinting, X-inactivation, and cancer (Schuettengruber et al., 2007).

In *Drosophila*, where the Polycomb system was first described, PcG and TrxG are specifically recruited on so-called Polycomb Response Element (PRE)/Trithorax Response Element (TRE) sequences, which are switchable memory DNA modules, with PcG or TrxG as their effectors (Schuettengruber et al., 2011). The mechanisms underlying PcG recruitment in mammals are still controversial, though some vertebrate PRE-like elements have recently been described. Interestingly, these retain features of *Drosophila* PREs including binding sites for DNA-binding of factors involved in PcG recruitment to PREs in *Drosophila* (Sing et al., 2009; Woo et al., 2010; Cuddapah et al., 2012). However, a defined role for mammalian homologs of PcG recruiters has not been established. Accordingly, additional mechanisms for PcG recruitment in mammals have been proposed. Several examples for a role of short and long ncRNAs in PcG recruitment in ES cells are available (Rinn et al., 2007; Zhao et al., 2008; Khalil et al., 2009; Gupta et al., 2010; Kanhere et al., 2010; Guil et al., 2012). Moreover, in mammals there is a strong correlation between PcG binding and CpG islands (Tanay et al., 2007; Ku et al., 2008; Mendenhall et al., 2010). In particular, non-methylated GC-rich sequences depleted of activating motifs have been shown to be sufficient for Polycomb recruitment in mammalian embryonic stem cells (Mendenhall et al., 2010).

Polycomb proteins form two major multiprotein complexes, Polycomb Repressive Complex 1 (PRC1) and 2 (PRC2). *Drosophila* PRC1 displays four core subunits: Polycomb (Pc), Polyhomeotic (Ph), Posterior sex combs (Psc), and Sex combs extra (Sce, also called dRing). PRC2 core subunits are Enhancer of zeste, E(z), Extra sex combs (Esc), Suppressor of zeste 12, Su(z)12, and the nucleosome-remodeling factor 55 (Nurf-55). In vertebrates, PRC1 and PRC2 are conserved in overall organization, but display a higher complexity in terms of subunits and interactions, so that their composition is cell type- and developmental stage-dependent (Kuzmichev et al., 2002, 2004, 2005; Gao et al., 2012).

Both PRC1 and PRC2 complexes retain an enzymatic activity. In PRC1, the RING domain containing protein dRing (Ring1B in vertebrates) is an E3 ubiquitin ligase mediating the ubiquitination of lysine 119 on histone H2A, which has been suggested to induce chromatin compaction and inhibit transcription elongation (De Napoles et al., 2004; Wang et al., 2004). Nevertheless, in the case of Ring1B the requirement of the enzymatic activity for chromatin compaction was recently challenged (Eskeland et al., 2010). PRC2 catalyzes the di-methylation and tri-methylation of histone H3 at lysine 27 (H3K27me2/me3). The catalytic subunit of PRC2, E(z) in flies, Enhancer of zeste homologs 1/2 (Ezh1/Ezh2) in vertebrates, contains the SET histone methyltransferase domain (Morey and Helin, 2010). Importantly, for its activity, E(z) requires the binding of two other PRC2 core components, Su(z)12/suppressor of zeste 12 (Suz12), and Esc/embryonic ectoderm development (Eed) (Morey and Helin, 2010).

H3K27me3 is a fundamental histone mark (hallmark) of Polycomb binding. Frequently, H3K27me3 is spread out to broad regions marking large PcG domains allowing for PREs-mediated repression several tens of kilobases away from target genes (Schuettengruber et al., 2007; Morey and Helin, 2010). H3K27me3 also represents a docking site recognized by PC (Cbx in vertebrates) contained in the PRC1 complex. Based on this, a sequential PRC2, PRC1 recruitment has been proposed (Cao et al., 2002). Nevertheless, it was recently shown that PRC1 recruitment to target genes in mammals can be also independent from PRC2 (Gao et al., 2012; Tavares et al., 2012).

### Polycomb and repeats

Polycomb-associated histone marks are prevalent in the mammalian genome. Quantitative mass spectrometry studies reported that up to 70% of histone H3 carries the PRC2 histone marks H3K27me2 or me3 (Peters et al., 2003; Schoeftner et al., 2006). However, genes and known functional elements comprise only up to 10% of the mammalian genome (Pheasant and Mattick, 2007), while over two-thirds of the remaining part is composed of repetitive elements (De Koning et al., 2011). Hence, this simple observation raises interesting questions about the possible acquirement of novel functions by the PcG proteins along with evolution, involving the non-coding fraction of the mammalian DNA.

Several reports show the presence of Polycomb repressive histone marks on repetitive elements. Initially, PcG silencing on repeats was described as a compensatory mechanism upon loss of H3K9me3 repression in pericentric DNA, where H3K27me1 was converted into H3K27me3 (Peters et al., 2003). More recently, the characterization of the epigenetic pattern of ERV-L and MaLR retrotransposons revealed that they are marked by H3K27me3 (Day et al., 2010), and importantly, a crucial role for ERV-L retrotransposons in embryo totipotency and development has been described (Macfarlan et al., 2011, 2012). At the very early two-cell stage, the murine endogenous retroviral elements ERVL (MuERV-L) are transiently de-repressed (Kigami et al., 2003). Their expression is significant, as it represents 3% of the total transcriptional output, and it is very sharply regulated in time, as it is specific for the developmental stage of the embryo where blastomeres are still totipotent (Svoboda et al., 2004).

Importantly, ERVL transcripts represent a source of regulatory elements which is co-opted by cellular genes to co-regulate their cell stage-specific expression (Macfarlan et al., 2012). In this process, more than 25% of MuERV-L copies are activated and 307 protein-coding genes generate 626 different chimeric transcripts with MuERV-L elements. Among the genes that use alternative MuERV-L-LTR promoters to initiate their transcription, there are transcription factors like Gata-4, which is involved in lineage determination and embryo development (Soudais et al., 1995) and is a known PcG target (Tiwari et al., 2008). Remarkably, MuERV-L expression is regulated by histone modifications like H3K4me3, the active histone mark typical of TrxG proteins (Schuettengruber et al., 2011). In fact, in the absence of the H3K4me3 demethylase LSD1/KDM1A, which is critical for the H3K4/H3K27 methylation balance in human ES cells (Adamo et al., 2011), MuERV-L/MERVL becomes overexpressed and embryonic development arrests at gastrulation (Macfarlan et al., 2011). Given that Polycomb and Trithorax are the major players in development and ERV-L is repressed via PcG mediated-silencing (Day et al., 2010), it is tempting to speculate that MuERV-L retrotransposons undergo a Polycomb/Trithorax regulation, with Polycomb mediating their repression and Trithorax their spatiotemporal-specific up-regulation in order to drive cell-fate specification.

A direct link between Polycomb and repeats-mediated silencing has been recently reported (Leeb et al., 2010). This work, in fact, not only identified both murine leukemia virus (MLV) and IAP retroelements as targets of Polycomb complexes, but also performed the first PcG loss-of-function study in a genomic repeat contest. Indeed, upon double knock out of key PRC1 and PRC2 components, Leeb et al. observed a strong increase in expression of LTR retrotransposons, which in turn provoked their active mobilization (Leeb et al., 2010). In particular, both MLV and IAP elements were found strongly de-repressed in ES cells double null for the Polycomb proteins Eed and Ring1B when compared to both wild type and single KO cells. Importantly, loss of binding of Polycomb complexes on MLV and the subsequent de-repression of these elements was associated with a considerable increase in MLV mobilization (Leeb et al., 2010). Similarly, Eed^−/−^ Ring1B^−/−^ ES cells showed IAP de-repression, that was associated with reduced levels of DNA methylation on IAP repeats in the double KO and Eed^−/−^ ES cells, in agreement with a previous report about repressive function on IAP retroelements of DNA methylation (Walsh et al., 1998). Hence, this work once again showed a redundancy in the mechanisms of repeat silencing, similarly to that previously reported for other repressive histone marks (Peters et al., 2003; Svoboda et al., 2004). Both PRC1 and PRC2 complexes, in fact, are recruited in parallel for LTR PcG-mediated silencing, as the single KO produced only a partial effect of de-repression (Leeb et al., 2010), thus suggesting that mechanisms of retrotransposon repression act redundantly even when mediated by the Polycomb machinery. Based on these results, it was suggested that genomic repeats, for their intrinsic feature of being present in several copies in the genome, could constitute binding platforms for mammalian PcG complexes (Leeb et al., 2010). Notably, epigenetic silencing of transgenes present in multiple copies has been already described in mice (Garrick et al., 1998; Festenstein et al., 1999; Hiragami and Festenstein, 2005) and it is well-established that proximity of DNA binding sites encourages cooperation among transcription factors (Amouyal et al., 1998; Amouyal, 2007).

Since the greatest proportion of Polycomb-mediated chromatin modifications is located in non-genic regions, a loss of PcG activity would need to be considered not only for its specific effect on Polycomb targets, but also for its possible effects on genome stability.

### nCRNAs in a polycomb and repeat landscape

**Figure 1:**
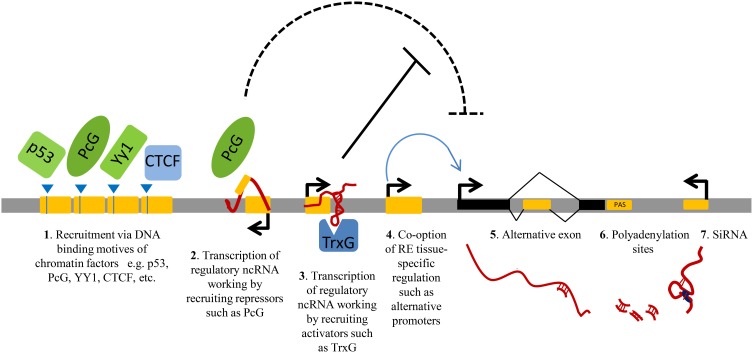
**A schematic view of the principal gene-regulatory functions of repeats.** Repetitive elements (RE, represented as yellow modules) have an impact on gene expression by providing DNA binding sites for transcription factors or chromatin regulators **(1)**; upon transcription, by generating regulatory non-protein-coding RNAs (ncRNAs) involved in gene silencing **(2)** or gene activation **(3)**, for example via direct recruitment of repressors/activators such as Polycomb (PcG) and Trithorax (TrxG) **(2–3)**. Repeat sequences can also contribute to gene transcription by providing alternative promoters **(4)**; alternative exons **(5)**; polyadenylation sites (PAS, **6**) or they can influence the stability of other transcripts via RNA interference (RNAi) by producing short double-stranded RNAs (dsRNAs) **(7)**.

Repeats can be specifically transcribed. Around 6–30% of the total amount of transcripts in mammalian cells initiates within repetitive elements and their expression is frequently tissue-specific (Faulkner et al., 2009). Recent studies show that repeats play central roles in regulating gene expression at multiple levels (Norris et al., 1995; Speek, 2001; Faulkner and Carninci, 2009; Kaneko et al., 2011; Shen et al., 2011). Repetitive elements may regulate the expression of nearby protein-coding genes by providing tissue-specific promoters or enhancers (Speek, 2001; Conley et al., 2008; Faulkner et al., 2009); they can be co-opted to generate alternative exons (Zhang and Chasin, 2006); they can modulate the abundance of gene products, for example through generation of ncRNAs, working *in trans* or *in cis*, either enhancing (by anti-silencing) or reducing (by transcriptional interference) their expression (Allen et al., 2004); or they can produce short ncRNAs exploited by RNAi machinery (Ghildiyal et al., 2008; Watanabe et al., 2008; Faulkner and Carninci, 2009) (Figure 1).

If the regulatory functions are combined with the ability of retrotransposons to mobilize upon de-repression or of tandem repeats to rearrange during meiotic division, the scenario becomes even more complex, as novel insertions of mobile repetitive elements or change in tandem repeat copy number may modify the chromatin structure (Lunyak et al., 2007) and the gene regulation of nearby genes (Cabianca et al., 2012).

Important examples of the interplay between Polycomb, repeats and ncRNAs in normal physiology and in disease are illustrated in the following sections.

### X-inactivation

X-inactivation, the process that leads to the silencing of one X chromosome in mammalian female cells, represents one of the most striking examples of long-range chromosomal regulation involving ncRNAs, Polycomb-mediating silencing and DNA repeats (Hall and Lawrence, 2010). In mammals, a large non-coding RNA named *Xist* “paints” the X-chromosome *in cis* (Brockdorff et al., 1992; Brown et al., 1992; Clemson et al., 2006; Chow et al., 2007) and induces a silencing cascade repressing the whole chromosome territory (Hall and Lawrence, 2003; Heard and Disteche, 2006). *Xist* works by recruiting PRC1, PRC2 and their respective histone marks (Leeb and Wutz, 2007) to the core of the inactive X chromosome, which contains genomic repeats (Chaumeil et al., 2006; Clemson et al., 2006). Besides local changes, a higher-order remodeling of the chromatin architecture takes place, thus producing the well-known silent core corresponding to the DAPI-dense Barr Body, which resides in the heterochromatic compartment at the nuclear or nucleolar periphery (Clemson et al., 2006).

Different classes of repeats play their roles in X-inactivation. Common repeats, like LINE-1 and Alu, participate structurally in the formation of the heterochromatic inner core of the Xi DNA territory (Hall and Lawrence, 2010), whereas a role for the euchromatic DXZ4 macrosatellite locus in Xi chromosome has been suggested (Chadwick, 2008). Moreover, the *Xist* ncRNA contains several tandem repeats termed A, B, C, D, E, and F (Hendrich et al., 1997; Nesterova et al., 2001; Yen et al., 2007; Horvath et al., 2011). Repeat A, with its conserved sequence and tetra-loop structure (Duszczyk et al., 2011), is essential for Polycomb-mediated silencing of X-linked genes (Wutz et al., 2002; Zhao et al., 2008). In fact, in the future Xi chromosome, PRC2 is initially recruited by the 1.6 kb *RepA* ncRNA, which is directly bound by the PRC2 subunit Ezh2. The *RepA*/PRC2 interaction enables the full-length *Xist* induction and thus the spreading of the *Xist* ncRNA and PcG silencing on the whole Xi chromosome (Zhao et al., 2008). The *RepA* region is the primary target of PcG binding also within the 17 kb full-length ncRNA *Xist* (Zhao et al., 2008), and indeed in *RepA* mutants, *Xist* recruits 80–90% less PRC2 (Kohlmaier et al., 2004).

The antisense 40 kb *Tsix* ncRNA is able to inhibit the *RepA*/Ezh2 interaction, probably by competing with *Xist* for PRC2 binding (Zhao et al., 2008). In pre-XCI cells, *Tsix* keeps in check the state of both X chromosomes and only a few molecules of *Xist* are transcribed (Zhao et al., 2008). When cell differentiation triggers dosage compensation, another regulatory ncRNA named *Jpx* becomes actively transcribed from the *Xist* loci of both X chromosomes, thus supplying the required activator for high-level *Xist* expression (Tian et al., 2010). In the future Xi, *Tsix* is now down-regulated, hence producing a permissive state for *Xist* induction, whereas, in the future Xa, the levels of *Tsix* continue titrating away PcG from *RepA*, thus maintaining blocked the repressive cascade (Zhao et al., 2008).

**Figure 2:**
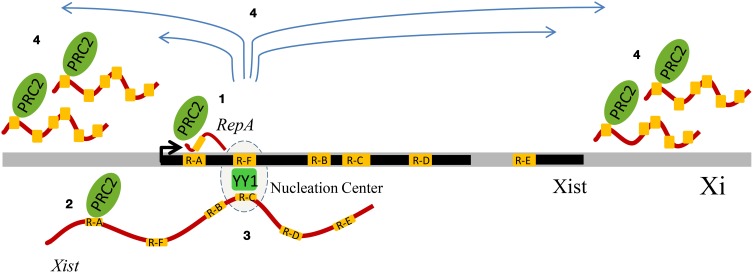
**Schematic summary of the influence of genomic repeats on X-chromosome inactivation (XCI).** The *Xist* DNA locus displays tandem repeats (visualized in yellow) and generates multiple transcripts (such as *RepA* and *Xist*), whose contribution to XCI involves binding to Polycomb Repressive Complex 2 (PRC2) and YY1, which has also been associated to Polycomb. Four sequential events of XCI are represented. During the initiation phase of XCI, the Repeat A (R-A) region of the ncRNA *RepA* recruits PRC2, creating the conditions for the production of the full-length Xist RNA **(1)**. *Xist* co-transcriptionally binds PRC2 via its R-A region, and it is loaded onto chromatin **(2)**. YY1 functions as a bridge and anchors *Xist in cis*, by binding both *Xist* RNA and DNA, respectively via their Repeat C (R-C) and Repeat F (R-F) regions **(3)**. *Xist* RNA, first bound only on the nucleation center, spreads *in cis* and recruits PRC2, thus mediating the X-chromosome inactivation **(4)**.

Another important *Xist* repeat is Repeat C, a C-rich sequence, specific of *Xist* and highly conserved, which is important for *Xist* localization on the inactive X chromosome (Memili et al., 2001; Sarma et al., 2010). A recent report provided an important role for another repeat of the *Xist* locus, Repeat F (Jeon and Lee, 2011). This region, characterized by the presence of CTCF and YY1 binding sites, is bound by YY1, which with its multiple zinc fingers is able to bind both DNA and RNA at the same time. YY1 bridges the *Xist* ncRNA via Repeat C (Sarma et al., 2010), and the X chromosome, via the Repeat F region. Overall, X inactivation provides a strong argument for an important physiological interplay between repeats, Polycomb, and ncRNAs (Figure 2).

### FSHD muscular dystrophy

FSHD (OMIM 158900) is a genetic disorder of particular interest for the atypical interactions between genetic and epigenetic players, which both contribute to the etiology of the disease (Neguembor and Gabellini, 2010). FSHD is an autosomal dominant disease and for more than 20 years it has been known to be associated with reduction in copy number of a macrosatellite repeat (called D4Z4) mapping to the subtelomeric 4q35 region (Wijmenga et al., 1990, 1991, 1992; Van Deutekom et al., 1993). Also, it has been known for a decade that D4Z4 deletions cause de-repression of genes located nearby (Gabellini et al., 2002). Nevertheless, the molecular understanding of the D4Z4 repeat mechanism of action was only recently provided (Cabianca et al., 2012).

Each D4Z4 unit is extremely GC rich, containing a sequence nearly identical to the consensus motif of *Drosophila* PREs and several putative DNA binding sites for factors which are Polycomb recruiters in *Drosophila*, such as YY1 and GAGA factor (Mihaly et al., 1998; Busturia et al., 2001; Mishra et al., 2001; Gabellini et al., 2002; Cabianca et al., 2012). Accordingly, in healthy subjects the D4Z4 tandem array is extensively bound by PRC1 and PRC2 and displays enrichment for the typical PcG-associated repressive histone marks H2AK119Ub and H3K27me3. The region is also bound by proteins associated to Polycomb recruitment in mammals like Jarid2 (Peng et al., 2009; Shen et al., 2009; Landeira et al., 2010; Li et al., 2010; Pasini et al., 2010) or homologs of PcG recruiters in *Drosophila* (YY1, HMGB2, c-Krox/Th-POK; vertebrate fly homologs Pho, Dsp1, GAGA factor, respectively) (Busturia et al., 2001; Mishra et al., 2001; Gabellini et al., 2002; Dejardin et al., 2005; Matharu et al., 2010). Finally, the repeats array also shows enrichment for the Polycomb-associated histone variant macroH2A (Buschbeck et al., 2009).

**Figure 3:**
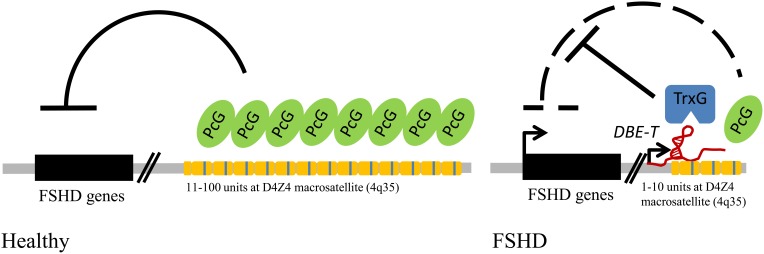
**FSHD muscular dystrophy links repetitive elements, Polycomb proteins, and ncRNAs in a human genetic disease.** Model for FSHD molecular pathogenesis: in healthy individuals the repetitive elements (yellow modules) of the D4Z4 macrosatellite at 4q35 are bound by Polycomb (PcG) proteins, which mediate gene repression; in FSHD patients the shortening below the threshold of 11 copies generates an epigenetic remodeling of the locus, sustained by a long non-coding RNA (*DBE-T*), and the recruitment of Trithorax (TrxG) proteins, which leads to up-regulation of disease candidate genes.

Importantly, D4Z4 is able to initiate PcG recruitment to ectopic sites and mediate copy number-dependent repression of gene expression, typical features of *Drosophila* PREs (Gabellini et al., 2002; Cabianca et al., 2012). In FSHD patients, the reduction in D4Z4 copy number is associated with a reduction in PcG silencing. This allows for the production of a long, chromatin-associated ncRNA: *DBE-T*. *DBE-T* works *in cis* by directly recruiting the TrxG protein ASH1L to the 4q35 locus. This leads to a structural and epigenetic remodeling of the FSHD locus, toward a more active chromatin state, which is responsible for the de-repression of 4q35 genes. Altogether, FSHD constitutes an important example of the relevance of DNA repeats, Polycomb and ncRNAs in human genetic diseases (Figure 3).

### Repetitive elements and chromatin organization in the 3D nuclear space

In general, nuclear organization of chromatin reflects its active or inactive state. Euchromatin occupies the internal nucleoplasm, whereas heterochromatin preferentially localizes at the nuclear and nucleolar periphery (Kosak et al., 2002; Shopland et al., 2003). Accordingly, repetitive elements can also localize differently. For example, pericentromeric satellite repeats are usually confined to the heterochromatic domains of the nuclear periphery whereas telomeres of human chromosomes usually reside in the internal compartment (Tam et al., 2004). There are important exceptions; the FSHD-associated 4q35 telomere behaves differentially, being usually associated to the nuclear periphery (Masny et al., 2004; Tam et al., 2004).

The nuclear machineries are not uniformly distributed in the nucleoplasm, but are organized in functional sub-compartments, so-called “factories” or “hubs” (Lamond and Spector, 2003; Hall et al., 2006; Meaburn and Misteli, 2007). In fact, by staining for a particular key factor of important nuclear processes (like transcription, RNA processing, replication, or DNA repair), a number of discrete structures appear in the nucleus, which result from the local concentration of proteins involved in specific nuclear processes. For example, “transcription factories” have been described and different genes, localized on distant chromosomal loci, can associate to the same active foci to be co-transcribed (Osborne et al., 2004). For nuclear compartments, patterns of distribution in the nucleus, characteristic of the different cell type or differentiation state, can be recognized (Lanctot et al., 2007). However, it is still an open question whether a fragment of DNA needs to be primarily attracted to one of these nuclear compartments in order to be functionally processed, or if the specific machinery can also activate elsewhere in the nucleus but needs to reach these structures for a higher efficiency.

Polycomb proteins and associated histone marks reside in discrete nuclear structures called Polycomb bodies, co-localizing with stably repressed homeotic genes (Messmer et al., 1992; Buchenau et al., 1998; Grimaud et al., 2006; Ferraiuolo et al., 2010; Bantignies et al., 2011). These repressive chromatin hubs are composed of chromatin loops involving PcG-bound regulatory elements and promoters of PcG target genes (Cleard et al., 2006; Comet et al., 2011). Hence not only events associated with gene activation, but also those associated with gene repression, including the ones involving Polycomb proteins, can localize on discrete foci, where long-range interactions take place.

**Figure 4:**
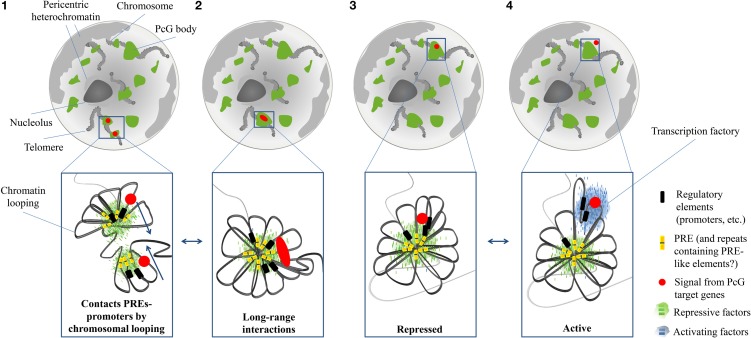
**Schematic representation of the distribution and dynamics of Polycomb (PcG) bodies within the nucleus.** PcG bodies (green) are hubs where, by chromatin looping, Polycomb Response Elements (PREs; yellow bars) closely interact with promoters (black bars) of PcG target genes (red circles), and where PcG proteins and other repressive factors (small green bars) accumulate, thanks to the binding to PREs **(1)**. PcG-bound elements and promoters are able to engage long-range chromatin interactions, so that two different PcG bodies cluster into the same structure. Red oval symbolizes co-localization of independent signals from remote PcG target genes **(2)**. Chromatin loops can adopt different spatial conformations, so that PcG target genes can be retained or displaced from PcG bodies, depending on their transcriptional state. In the repressed state, a condensed structure tightens the interactions among all PcG-bound elements **(3)**. When a stimulus activates the transcription of a PcG target gene, its promoter loses the interaction with PREs, and co-localizes with activators (small blue bars), within transcription factories (blue cloud) **(4)**.

The organization of these structures in *Drosophila* starts at the level of PREs, the DNA modules recruiting Polycomb complexes (Muller and Kassis, 2006; Schuettengruber et al., 2007). As already discussed, the histone-methylation activity of the PRC2 complex spreads out on neighboring regions, marking large PcG domains. Hence, PcG silencing reaches target genes that are tens of kilobases distant from a PRE. Moreover, PREs tend to cluster in larger domains (Bantignies and Cavalli, 2011) (Figure 4).

As characterized by chromosome conformation capture experiments, long distance intra- and even inter-chromosomal interactions among PcG targets are established, thus producing a major level of chromatin organization in the 3D nuclear space (Lanzuolo et al., 2007; Terranova et al., 2008; Tiwari et al., 2008; Eskeland et al., 2010; Comet et al., 2011; Tolhuis et al., 2011). It has been proposed that these long-range contacts are mediated by ncRNAs (Rinn et al., 2007), insulators DNA element (Li et al., 2011) and RNAi machinery (Grimaud et al., 2006). On top of such a hierarchal organization of PcG domains are found the PcG bodies. PcG bodies differ in size and Polycomb intensity. In particular, PcG domains with a larger linear size display a higher content of Polycomb and generate bigger and more intense PcG bodies (Cheutin and Cavalli, 2012).

The discovery of PcG bodies raised questions about their function: are they merely the result of the accumulation of PcG proteins to clustered Polycomb domains, or is the formation of these “hubs” required for PcG silencing (Buchenau et al., 1998)? The fact that PcG proteins organize in such PcG bodies instead of being uniformly distributed in the nucleus is already an indication toward a functional role for these structures. Indeed, PcG-mediated gene silencing occurs within PcG bodies (Grimaud et al., 2006) and it has been proposed that the local concentration of PcG components and their target genes in PcG bodies may produce chromatin condensation (Terranova et al., 2008; Eskeland et al., 2010). Indeed, a correlation between repression of PcG targets and their localization in PcG bodies has been reported. For example, Fab-7, the PRE-containing region controlling the expression of the gene Abd-B, is found within PcG bodies when Abd-B is repressed, whereas it is outside the PcG bodies when Abd-B is expressed (Lanzuolo et al., 2007; Bantignies and Cavalli, 2011; Bantignies et al., 2011). Active genes are displaced from these repressive chromatin hubs not only in *Drosophila* but also in mammals. For example, the human GATA-4 locus, involving several PcG bound regions, shows a similar chromatin structure depending on its transcriptional state (Tiwari et al., 2008) (Figure 4).

As it primarily functions as a marker regulator of development, Polycomb accumulation, and thus the presence of PcG bodies are regulated during cell differentiation. Experiments of fluorescence recovery after photobleaching (FRAP) in both *Drosophila* and mammalian embryonic stem cells, showed a dynamic exchange of PcG proteins between PcG bodies and nucleoplasm (Ficz et al., 2005; Ren et al., 2008). In *Drosophila*, Polycomb starts accumulating in the nucleus during the early stages of development (stage 5), progressively increases and gets recruited to PcG bodies (stages 5–11), until it becomes stably associated with PcG bodies during late embryogenesis (Cheutin and Cavalli, 2012). To address the question of whether the formation of PcG bodies is the direct result of PcG binding to their targets or, on the contrary, PcG targets need to associate with PcG bodies in order to be repressed, *in vivo* live imaging approaches have been used to characterize the motion of PcG targets and PcG bodies in the nucleus. Interestingly, a motion away from PcG bodies from the nuclear periphery toward the nuclear interior, regulated by actin and nuclear myosin I, was observed immediately after inducing transcription (Chuang et al., 2006). Similarly to other chromatin domains, Polycomb bodies’ motion sensitively decreases upon differentiation, and shows similar kinetics, either fast but limited to volumes much smaller than chromosome territory occupancy, or slow but involving overall a higher level of nuclear structure (Cheutin and Cavalli, 2012).

Based on the fact that Polycomb is concentrated in PcG bodies by immunofluorescence and in repeats by chromatin immunoprecipitation, it could be hypothesized that genomic repeats which are Polycomb targets in mammals could functionally behave in a similar way to PREs and mediate association between Polycomb-regulated genes. In this view, Polycomb complexes and repetitive elements would play a role in the compartmentalization of the nucleus, establishing large chromatin domains where PcG target genes are efficiently repressed. Interestingly, it has been shown that the 3D organization of PcG target genes can influence PcG-mediated silencing. In *Drosophila* the deletion of Fab-7 perturbed the interaction between BX-C and ANT-C, producing mild effects on gene expression at distant Polycomb target genes. However, sensitized genetic backgrounds had to be used in order to observe homeotic phenotypes (Bantignies et al., 2011). Interestingly, in mammals structural alterations of repetitive sequences can affect long-range PcG-mediated silencing *in cis* (Cabianca et al., 2012). Moreover, deletions or mutations of genetic elements on one chromosome can affect expression of interacting genes *in trans* (Spilianakis et al., 2005; Ling et al., 2006).

Collectively, these considerations strongly indicate that investigation of the role of repetitive sequences in nuclear structural organization in mammals is an important topic for future research. This will require a significant operational and conceptual shift. Operationally, genome-wide approaches would have to be tailored to the analysis of repetitive sequences, which represents a serious bioinformatics challenge. Conceptually, investigators should take into consideration the biological relevance of the major component of the human genome, being aware that this could potentially change the understanding of how the nuclear processes work.

### Conflict of interest statement

The authors declare that the research was conducted in the absence of any commercial or financial relationships that could be construed as a potential conflict of interest.
